# Assessment of Psychosocial Determinants of Quality of Life Among Healthcare Professionals at a Tertiary Care Hospital in Chennai, India

**DOI:** 10.7759/cureus.101477

**Published:** 2026-01-13

**Authors:** Vishala Rao, Sindhu RSS, Jasmine Kavitha Washington, Minthami Sharon P, Ishwarya S

**Affiliations:** 1 Obstetrics and Gynecology, Sree Balaji Medical College and Hospital, Chennai, IND

**Keywords:** healthcare workers, occupational stress, psychosocial factors, quality of life (qol), work-life balance

## Abstract

Background: Work-related pressures among healthcare workers (HCWs) are substantial and can adversely affect their quality of life (QoL). Understanding the psychosocial and occupational factors influencing QoL is crucial for developing effective strategies to enhance the healthcare workforce's well-being and performance. This study aimed to assess the QoL of healthcare professionals at a tertiary care hospital in Chennai, India, and to examine the impact of occupational and psychological factors on their overall well-being.

Methods: A cross-sectional study was conducted among 242 healthcare professionals in a tertiary care hospital in Chennai (111 medical doctors, 131 nurses) using convenience sampling. Data were collected on sociodemographic characteristics, occupational variables (working hours, length of employment, workplace support), and QoL using standardized based on Perceived Stress Scale (PSS-10) for stress measurement, the Pittsburgh Sleep Quality Index (PSQI) for sleep quality, the Alcohol Use Disorders Identification Test (AUDIT) for alcohol consumption, and the Fagerström Test for Nicotine Dependence (FTND) for smoking behavior across four domains (physical health, psychological health, social relationships, and environmental health). Comparative analyses between subgroups were performed using independent t-tests, while Pearson correlation assessed relationships between occupational factors and QoL domains.

Results: Nurses reported significantly lower QoL scores than doctors across all domains (total QoL: 57.16 vs. 63.6; p < 0.001). Healthcare professionals working more than 48 hours per week had notably reduced QoL, particularly in social relationships (12.5 vs. 14.88) and physical health (13.14 vs. 15.11), with strong negative correlations observed (social: r = -0.577; physical: r = -0.492). Participants with more than one year of employment demonstrated higher QoL in physical (14.15 vs. 12.78) and psychological domains (12.83 vs. 11.56), suggesting adaptation and professional stability. Workplace support showed a modest positive association with psychological health (r = 0.179).

Conclusion: QoL among healthcare professionals is strongly influenced by occupation, working hours, employment duration, and workplace support. Nurses and those with longer working hours are particularly vulnerable to lower QoL. Interventions promoting work-life balance, workload management, mentorship, and mental health support are essential to enhance the well-being and professional satisfaction of healthcare personnel in tertiary care settings.

## Introduction

Healthcare workers (HCWs) play a vital role in maintaining the functionality and resilience of health systems, often working under highly demanding and stressful conditions that can profoundly affect their quality of life (QoL). According to the World Health Organization (WHO), QoL refers to an individual's perception of their position in life within the context of their culture, value systems, personal goals, expectations, and concerns [1-4]. In the context of healthcare professionals, QoL is influenced by a broad spectrum of psychosocial and occupational factors such as workload, emotional strain, workplace environment, support systems, and lifestyle habits. The World Health Organization Quality of Life (WHOQOL) framework operationalizes QoL across four core domains: physical health (pain, energy, sleep, mobility), psychological health (positive feelings, self-esteem, cognition), social relationships (personal relationships, social support), and environmental factors (financial resources, healthcare access, safety, home environment). Understanding these determinants is essential for formulating strategies that promote the well-being of HCWs, which, in turn, enhances patient care outcomes and the overall efficiency of healthcare delivery systems [5].

The nature of healthcare work demands sustained emotional engagement, rapid decision-making, and long working hours - often under physically and psychologically challenging circumstances. Continuous exposure to stressful environments, infectious diseases, and ethical dilemmas places HCWs at risk for occupational burnout, a condition characterized by emotional exhaustion, depersonalization, and reduced personal accomplishment [6]. Studies have consistently shown that high workloads, insufficient rest, and a lack of work-life balance contribute to mental distress, diminished job satisfaction, and reduced QoL among healthcare professionals. Physicians and nurses, in particular, face significant psychological burdens due to the intense nature of their duties, which may lead to anxiety, depression, and sleep disturbances [7]. The compounding effect of chronic stress not only undermines individual well-being but can also compromise clinical performance and patient safety.

Workplace psychosocial factors have a critical impact on HCWs’ perceived QoL. Organizational support, both from supervisors and peers, has been identified as a protective factor against stress and burnout. A positive and cooperative work culture fosters emotional resilience, strengthens coping mechanisms, and enhances overall job satisfaction [8,9]. Conversely, lack of institutional or peer support often leads to emotional exhaustion, reduced motivation, and lower productivity. Supportive supervision, fair workload distribution, and team cohesion are, therefore, integral to improving morale and maintaining sustainable workforce engagement. Additionally, personal lifestyle habits, such as smoking, alcohol consumption, diet, and sleep quality, play a pivotal role in determining QoL. Poor sleep hygiene, which is common among shift-working HCWs, contributes to cognitive decline, impaired concentration, and heightened vulnerability to psychological disorders [10].

The relationship between length of employment and QoL is another crucial dimension explored in recent literature. Early-career HCWs, particularly those with less than a year of experience, may face heightened stress due to steep learning curves, adaptation challenges, and insufficient coping mechanisms [11]. In contrast, experienced HCWs often exhibit greater resilience and emotional stability; however, long-term exposure to occupational strain can result in chronic fatigue and cumulative psychological wear, which gradually erode QoL. Similarly, excessive working hours, particularly those exceeding 48 hours per week, are strongly correlated with elevated levels of stress, emotional exhaustion, and dissatisfaction [12]. Such findings underscore the importance of regulated work schedules and adequate rest periods in maintaining workforce health and performance.

Beyond workplace stressors, external psychosocial determinants also significantly influence HCWs’ well-being. Family responsibilities, financial strain, and social support networks are vital external factors that can either buffer or exacerbate occupational stress [13]. Many HCWs, especially those balancing dual roles at work and home, experience persistent tension that impedes relaxation and recovery. The inability to maintain a healthy work-life balance often leads to emotional burnout and deteriorating QoL. Furthermore, repeated exposure to patient suffering, medical errors, and traumatic clinical events can evoke psychological distress, guilt, and moral injury, further diminishing mental well-being [14].

Given the central role HCWs play in patient care, their well-being is an indispensable component of healthcare system quality. A decline in HCWs’ QoL not only affects their personal health but also influences patient satisfaction, safety outcomes, and institutional performance. Thus, systematic evaluation of QoL and its associated psychosocial determinants is essential. Identifying the key stressors and protective factors enables healthcare administrators to implement evidence-based interventions such as stress reduction programs, structured duty hours, wellness initiatives, and accessible mental health support systems [12].

Despite growing recognition of burnout and psychological distress among HCWs, systematic evidence on overall QoL and its psychosocial determinants remains limited, particularly in tertiary care settings where workload intensity, staffing constraints, and emotional demands are high. This study aims to comprehensively assess the QoL among HCWs in a tertiary care hospital and to analyze the psychosocial elements influencing it. By elucidating the interplay between occupational stressors, personal factors, and social support, this research seeks to provide actionable insights for policy formulation and institutional reforms that enhance the overall well-being and job satisfaction of healthcare professionals. Ultimately, improving the QoL of HCWs is not only an ethical and humanistic imperative but also a strategic investment in the sustainability and quality of healthcare delivery systems.

## Materials and methods

Study design and setting

A cross-sectional descriptive study was conducted to assess the QoL among healthcare professionals and to identify the psychological and occupational elements influencing it. The research was carried out over three months (January to March 2025) at a tertiary care teaching hospital in Chennai, Tamil Nadu, India. The institution is a multidisciplinary healthcare center catering to a large population with extensive inpatient and outpatient services, making it an ideal setting to study diverse categories of healthcare professionals under varying levels of occupational exposure and workload.

The study design was chosen for its ability to capture a snapshot of the current status of QoL and its associated psychosocial determinants within a defined population. This approach allowed for the identification of prevalent trends and interrelationships between demographic, occupational, and psychological factors without manipulating any study variables. The cross-sectional design also enabled efficient collection of data within a limited timeframe, offering valuable insights that could inform institutional health promotion strategies and future longitudinal research.

Study population

The target population comprised doctors and nurses employed at the tertiary care hospital who were directly involved in patient care. This included professionals working across different departments such as general medicine, surgery, obstetrics and gynecology, pediatrics, anesthesia, and intensive care. Inclusion criteria were (1) currently employed healthcare professionals (physicians or nurses) with at least six months of service experience at the institution, and (2) willingness to participate voluntarily after obtaining informed consent.

To minimize bias and potential confounding factors, individuals with a known history of psychiatric illness, substance dependence, or ongoing psychological treatment were excluded from the study. This exclusion ensured that any reported variations in QoL were primarily related to occupational and psychosocial determinants rather than pre-existing mental health conditions. Participants who met criteria suggestive of alcohol dependence or high nicotine dependence on the Alcohol Use Disorders Identification Test (AUDIT) or the Fagerström Test for Nicotine Dependence (FTND) screening were excluded during analysis, in line with the predefined exclusion criteria. Individuals reporting occasional or low-risk use (AUDIT score <8; FTND indicating no or low dependence) were retained, as such use reflects real-world behavior and allowed assessment of its relationship with QoL outcomes.

The inclusion of both doctors and nurses allowed for a comparative perspective between different professional categories within the healthcare sector. Physicians and nurses often face differing stressors, workloads, and interpersonal interactions in their workplace, thus offering a comprehensive understanding of how job roles and responsibilities influence QoL outcomes.

Sample size determination

The sample size was calculated using the standard formula for estimating proportions in cross-sectional studies. For this study, the expected proportion of healthcare professionals experiencing compromised QoL was assumed to be 0.16 based on prior literature [15]. The desired precision was set at 0.05, and the two-sided Z value corresponding to a 95% confidence level was 1.96. Substituting these values yielded a minimum required sample size of 204 participants. However, to ensure sufficient statistical power and account for potential non-response or incomplete questionnaires, the final sample included 242 healthcare professionals.

Data collection tool

A pretested and standardized structured questionnaire was used as the primary tool for data collection (see Appendices). The instrument was designed to comprehensively assess various aspects of QoL, demographic variables, occupational characteristics, and psychosocial stressors.

The structured questionnaire used for this study comprised four major sections designed to comprehensively assess the QoL and associated psychosocial factors among healthcare professionals.

The first section, Demographic Profile, collected baseline information including age, gender, marital status, educational qualification, years of professional experience, and designation (doctor or nurse). The second section, Work-Related Characteristics, captured details regarding department of posting, average working hours per week, duty shift type (day, night, or rotational), and self-perceived workload intensity. The third section, Psychosocial Factors, assessed perceived social support, interpersonal workplace relationships, occupational stress, coping mechanisms, and lifestyle habits such as sleep quality, alcohol use, and smoking. Validated tools were used for these assessments, including the Perceived Stress Scale (PSS-10) for stress measurement, the Pittsburgh Sleep Quality Index (PSQI) for sleep quality, the AUDIT for alcohol consumption, and the FTND for smoking behavior [16-19]. All instruments were used in accordance with licensing agreements: PSS-10 requires registration with Mapi Trust, PSQI is free for non-commercial research, AUDIT is freely available from the World Health Organization (WHO), and FTND can be used under Inquisit Lab/Web licensing.

The fourth section, Quality of Life Assessment, measured self-reported satisfaction across physical health, psychological well-being, social relationships, and work-life balance using the WHOQOL-BREF instrument [20]. WHOQOL-BREF is available for research use with permission from the WHO. Responses were recorded on a 5-point Likert scale ranging from poor to excellent, allowing quantification of subjective well-being in multiple dimensions. The questionnaire was pilot-tested on a small group of HCWs from another institution to ensure clarity, cultural relevance, and internal consistency, and refinements were made based on feedback. The questionnaire showed satisfactory reliability, with Cronbach’s alpha values of 0.81 and 0.75.

The questionnaire was pilot-tested on a small group of HCWs from another institution to ensure clarity, cultural relevance, and internal consistency. Feedback from the pilot phase was incorporated to refine the phrasing and layout before final administration.

Data collection procedure

Participants were approached personally at their respective departments after obtaining permission from the institutional administration. The purpose and objectives of the study were clearly explained to each participant, and voluntary written informed consent was obtained prior to enrollment. Data collection was conducted in a confidential manner to ensure anonymity and promote honest responses.

The participants were instructed to complete the questionnaire independently without external influence. The average time taken to complete the questionnaire was approximately 20-25 minutes. Completed forms were collected on the same day to prevent data loss or unauthorized access. The anonymity of participants was preserved by using unique numerical identifiers instead of personal information. Throughout the data collection process, participants were encouraged to clarify any doubts regarding the questionnaire items. Regular monitoring was carried out by the principal investigator to ensure completeness, accuracy, and consistency of data entries.

Data management and quality control

Data from the completed questionnaires were manually checked for completeness and subsequently entered into a secure digital database. Double data entry was employed to minimize errors, and inconsistencies were cross-verified with the original forms. Data cleaning was performed before analysis to remove incomplete or invalid responses. Confidentiality was maintained throughout the process. Only the research team had access to the data, and all identifying information was excluded prior to analysis. The data were stored on password-protected computers to ensure information security and compliance with ethical standards.

Ethical considerations

Ethical clearance was obtained from the Institutional Human Ethics Committee (Sree Balaji Medical College and Hospital, Chennai, approval no. 002/SBMC/IHEC/2023/2294) before initiating the study. The research adhered to the principles outlined in the Declaration of Helsinki for studies involving human participants. All participants were informed about the study objectives, their right to refuse participation, and their ability to withdraw at any stage without any consequences. Written informed consent was obtained from each participant before administering the questionnaire. Confidentiality and anonymity were rigorously maintained throughout the study process. Data were used exclusively for academic and research purposes, and no personal identifiers were disclosed in the final analysis or publication.

Statistical analysis

All statistical analyses were performed using IBM SPSS Statistics for Windows, Version 26 (Released 2018; IBM Corp., Armonk, New York, United States). Descriptive statistics were applied to summarize the data. Continuous variables such as age and years of experience were expressed as mean ± standard deviation (SD), while categorical variables such as gender, profession, and work schedule were presented as frequency and percentage. To explore associations between independent variables (demographic, occupational, and psychosocial factors) and dependent variables (QoL domains), the Student’s t-test was used for continuous variables and the chi-square test for categorical comparisons. A p-value of <0.05 was considered statistically significant. Correlation and regression analyses were also conducted to identify predictors of lower QoL among healthcare professionals.

## Results

Among the 242 HCWs included in the study, the majority were aged 22-30 years (129; 53.3%), followed by 41-50 years (72; 29.8%), 31-40 years (37; 15.3%), and 51-60 years (4; 1.7%). Females constituted 172 (71.1%) of the participants, while males accounted for 70 (28.9%). In terms of occupation, 111 (45.9%) were doctors and 131 (54.1%) were nurses. Most participants had been employed for more than one year (197; 81.4%), and 149 (61.6%) reported working more than 48 hours per week. A majority received support from colleagues or staff (193; 79.8%), while 49 (20.2%) did not. Regarding lifestyle factors, 40 (16.5%) were smokers, and 93 (38.4%) consumed alcohol. Sleep duration of less than six hours was reported by 89 (36.8%) participants, whereas 153 (63.2%) had more than six hours of sleep daily (Table 1).

**Table 1:** Demographic and psychosocial characteristics of the study participants (n = 242)

Variable	Category	Frequency	Percent (%)
Age	22-30 years	129	53.3
	31-40 years	37	15.3
	41-50 years	72	29.8
	51-60 years	4	1.7
Gender	Male	70	28.9
	Female	172	71.1
Occupation	Doctor	111	45.9
	Nurse	131	54.1
Length of Employment	<1 year	45	18.6
	>1 year	197	81.4
Working Hours	<48 hours	93	38.4
	>48 hours	149	61.6
Support From Staff	Yes	193	79.8
	No	49	20.2
Smoking	Yes	40	16.5
	No	202	83.5
Alcohol	Yes	93	38.4
	No	149	61.6
Sleep	<6 hours	89	36.8
	>6 hours	153	63.2

The comparison of QoL scores between doctors and nurses revealed statistically significant differences across all domains. Doctors demonstrated higher mean scores than nurses in physical health (14.69 vs. 13.22; t = 6.25, p < 0.001), psychological health (13.63 vs. 11.71; t = 8.45, p < 0.001), social relationships (14.07 vs. 12.86; t = 5.98, p < 0.001), and environmental health (13.99 vs. 13.07; t = 4.32, p < 0.001). The overall QoL score was also significantly higher among doctors (63.6) compared to nurses (57.16; t = 7.56, p < 0.001), indicating better perceived well-being among doctors across physical, psychological, social, and environmental domains (Table 2).

**Table 2:** Comparison of quality of life (QoL) scores between doctors and nurses using independent samples t-test P < 0.05 is considered statistically significant.

Domain	Category	N	Mean	SD	SE Mean	Mean Difference	95% CI (Lower-Upper)	t-value	p-value
Physical Health	Doctor	111	14.69	1.75	0.17	1.47	1.01-1.93	6.25	<0.001
	Nurse	131	13.22	1.86	0.16				
Psychological Health	Doctor	111	13.63	1.29	0.12	1.92	1.57-2.27	8.45	<0.001
	Nurse	131	11.71	1.44	0.13				
Social Relationships	Doctor	111	14.07	1.90	0.18	1.21	0.72-1.70	5.98	<0.001
	Nurse	131	12.86	1.94	0.17				
Environmental Health	Doctor	111	13.99	1.59	0.15	0.92	0.51-1.34	4.32	<0.001
	Nurse	131	13.07	1.67	0.15				
Total QoL Score	Doctor	111	63.60	6.20	0.59	6.44	4.77-8.12	7.56	<0.001
	Nurse	131	57.16	6.92	0.61				

The comparison of QoL domains between HCWs with different working hours revealed significantly higher QoL scores among those working less than 48 hours per week across all domains. Participants with shorter working hours reported better physical health (mean 15.11 vs. 13.14; t = 8.75, p < 0.001), psychological health (13.27 vs. 12.17; t = 5.25, p < 0.001), social relationships (14.88 vs. 12.50; t = 10.96, p < 0.001), and environmental health (14.38 vs. 12.94; t = 7.06, p < 0.001). The total QoL score was also significantly higher among those working less than 48 hours (64.81 vs. 57.19; t = 9.12, p < 0.001), indicating that prolonged working hours are associated with poorer overall well-being among healthcare professionals (Table 3).

**Table 3:** Comparison of quality of life (QoL) domains by working hours among study participants using independent samples t-test P < 0.05 is considered statistically significant.

QoL Domain	Working Hours	N	Mean	Std. Deviation	Std. Error Mean	Mean Difference	95% CI (Lower-Upper)	t-value	p-value
Physical Health	<48 Hours	93	15.11	1.83	0.19	1.97	1.52-2.41	8.75	<0.001
	>48 Hours	149	13.14	1.62	0.13				
Psychological Health	<48 Hours	93	13.27	1.33	0.14	1.10	0.69-1.51	5.25	<0.001
	>48 Hours	149	12.17	1.73	0.14				
Social Relationships	<48 Hours	93	14.88	1.48	0.15	2.38	1.95-2.81	10.96	<0.001
	>48 Hours	149	12.50	1.74	0.14				
Environmental Health	<48 Hours	93	14.38	1.41	0.15	1.44	1.04-1.84	7.06	<0.001
	>48 Hours	149	12.94	1.62	0.13				
Total QoL Score	<48 Hours	93	64.81	5.97	0.62	7.62	5.97-9.27	9.12	<0.001
	>48 Hours	149	57.19	6.54	0.54				

Table 4 compares the QoL domains among healthcare professionals based on their length of employment. Participants with more than one year of employment reported significantly higher mean scores across all QoL domains compared to those employed for less than one year. The mean physical health score was 14.15 ± 1.88 for long-term employees versus 12.78 ± 1.89 for short-term employees (t = -4.44, p < 0.001). Similar significant differences were observed for psychological health (t = -4.82, p < 0.001), social relationships (t = -4.60, p < 0.001), and environmental health (t = -3.71, p < 0.001). Overall, total QoL was markedly better among those with longer employment duration (61.26 ± 6.76 vs. 55.11 ± 7.68; t = -5.36, p < 0.001), suggesting that experience and workplace adaptation positively influence well-being.

**Table 4:** Comparison of quality of life (QoL) domains by length of employment among study participants using independent samples t-test P < 0.05 is considered statistically significant.

QoL Domain	Length of Employment	N	Mean	Std. Deviation	Std. Error Mean	Mean Difference	Std. Error Difference	95% CI Lower	95% CI Upper	t-value	p-value
Physical Health	<1 Year	45	12.78	1.894	0.282	-1.375	0.310	-1.986	-0.763	-4.44	<0.001
	>1 Year	197	14.15	1.876	0.134						
Psychological Health	<1 Year	45	11.56	1.589	0.237	-1.272	0.264	-1.792	-0.752	-4.82	<0.001
	>1 Year	197	12.83	1.601	0.114						
Social Relationships	<1 Year	45	12.22	2.275	0.339	-1.468	0.319	-2.096	-0.840	-4.60	<0.001
	>1 Year	197	13.69	1.844	0.131						
Environmental Health	<1 Year	45	12.67	1.651	0.246	-1.014	0.273	-1.550	-0.477	-3.71	<0.001
	>1 Year	197	13.68	1.649	0.117						
Total QoL Score	<1 Year	45	55.11	7.679	1.145	-6.148	1.146	-8.406	-3.890	-5.36	<0.001
	>1 Year	197	61.26	6.760	0.482						

The correlation analysis reveals several significant associations among occupational and workplace factors with QoL domains. Occupation showed significant negative correlations with physical health (r = -0.377, p < 0.001), psychological health (r = -0.574, p < 0.001), social relationships (r = -0.301, p < 0.001), environmental health (r = -0.272, p < 0.001), length of employment (r = -0.333, p < 0.001), and working hours (r = 0.330, p < 0.001), but not with support from staff (r = -0.031, p = 0.626). Physical health was positively correlated with psychological health (r = 0.646, p < 0.001), social relationships (r = 0.752, p < 0.001), and environmental health (r = 0.809, p < 0.001), while negatively correlated with working hours (r = -0.492, p < 0.001). Psychological health was positively associated with social relationships (r = 0.546, p < 0.001) and environmental health (r = 0.547, p < 0.001), and negatively with working hours (r = -0.321, p < 0.001). Social relationships correlated positively with environmental health (r = 0.812, p < 0.001) and length of employment (r = 0.285, p < 0.001), but negatively with working hours (r = -0.577, p < 0.001). Environmental health showed a positive correlation with length of employment (r = 0.233, p < 0.001) and a negative correlation with working hours (r = -0.414, p < 0.001). Length of employment was negatively associated with support from staff (r = -0.156, p = 0.015), while working hours and support from staff were not significantly correlated (r = -0.004, p = 0.956). Overall, these findings indicate that occupation, working hours, and employment duration significantly influence multiple QoL domains among healthcare professionals (Table 5).

**Table 5:** Correlations between quality of life (QoL) domains and occupational and psychosocial characteristics among study participants (Pearson’s correlation analysis) P < 0.05 is considered statistically significant.

Variables	Occupation	Physical Health	Psychological Health	Social Relationships	Environmental Health	Length of Employment	Working Hours	Support From Staff
Occupation	1	-0.377 (p<0.001)	-0.574 (p<0.001)	-0.301 (p<0.001)	-0.272 (p<0.001)	-0.333 (p<0.001)	0.330 (p<0.001)	-0.031 (p=0.626)
Physical Health	-0.377 (p<0.001)	1	0.646 (p<0.001)	0.752 (p<0.001)	0.809 (p<0.001)	0.275 (p<0.001)	-0.492 (p<0.001)	0.027 (p=0.679)
Psychological Health	-0.574 (p<0.001)	0.646 (p<0.001)	1	0.546 (p<0.001)	0.547 (p<0.001)	0.297 (p<0.001)	-0.321 (p<0.001)	0.179 (p=0.005)
Social Relationships	-0.301 (p<0.001)	0.752 (p<0.001)	0.546 (p<0.001)	1	0.812 (p<0.001)	0.285 (p<0.001)	-0.577 (p<0.001)	-0.018 (p=0.784)
Environmental Health	-0.272 (p<0.001)	0.809 (p<0.001)	0.547 (p<0.001)	0.812 (p<0.001)	1	0.233 (p<0.001)	-0.414 (p<0.001)	0.030 (p=0.644)
Length of Employment	-0.333 (p<0.001)	0.275 (p<0.001)	0.297 (p<0.001)	0.285 (p<0.001)	0.233 (p<0.001)	1	-0.050 (p=0.438)	-0.156 (p=0.015)
Working Hours	0.330 (p<0.001)	-0.492 (p<0.001)	-0.321 (p<0.001)	-0.577 (p<0.001)	-0.414 (p<0.001)	-0.050 (p=0.438)	1	-0.004 (p=0.956)
Support From Staff	-0.031 (p=0.626)	0.027 (p=0.679)	0.179 (p=0.005)	-0.018 (p=0.784)	0.030 (p=0.644)	-0.156 (p=0.015)	-0.004 (p=0.956)	1

## Discussion

This study evaluated the QoL among healthcare professionals in a tertiary care hospital, examining the influence of occupation, working hours, length of employment, and workplace support. Nurses consistently reported lower scores than doctors across all domains of QoL, including physical health, psychological health, social relationships, and environmental health. These findings align with Kumar et al. (2018), who highlighted occupational stress as a key determinant of QoL, and that nurses frequently experience higher stress due to patient-centered responsibilities and emotionally demanding work environments [21].

Working hours had a significant impact on QoL. Participants working fewer than 48 hours per week reported higher overall QoL scores (64.81 vs. 57.19), with social relationships (14.88 vs. 12.5) and physical health (15.11 vs. 13.14) being particularly affected. Longer shifts are known to be associated with burnout, fatigue, and poor health outcomes among HCWs [15]. Correlation analysis confirmed negative associations between working hours and social health (r = -0.577) as well as physical health (r = -0.492), highlighting the adverse effects of extended work schedules. Length of employment also influenced QoL. HCWs with more than one year of experience had higher scores across all domains, especially physical (14.15 vs. 12.78) and psychological health (12.83 vs. 11.56), suggesting that longer tenure allows professionals to develop coping mechanisms, achieve stability, and adapt to occupational demands [22]. This aligns with Asante et al. (2019), who observed that experienced HCWs were better able to manage stress, resulting in improved QoL [22].

Workplace support, though modest, had a positive effect on psychological health (r = 0.179). Consistent with Quinones-Rozo et al. (2024), this demonstrates that collegial support, effective leadership, and structured mentoring contribute to mental well-being [23]. Participants reporting adequate support at work had higher psychological health scores, emphasizing the importance of institutional systems that foster professional support and guidance. Comparisons between doctors and nurses revealed substantial disparities in QoL. Nurses scored lower in all domains, which aligns with qualitative findings by Nalini et al. (2024) and Shivani et al. (2023), who highlighted heightened occupational and psychological stress among nurses, particularly in high-intensity settings such as oncology and neonatal intensive care units (NICUs) [24,25]. The high patient load, emotional labor, and frequent shift demands likely contribute to the reduced QoL observed among nurses in this study.

Our findings support the observation of Asante et al. (2019) that healthcare professionals often report low QoL, especially in social and physical health domains [22]. Extended working hours were significantly associated with poorer social interactions and physical health outcomes, underscoring the need for institutional interventions to regulate workload and prevent burnout. Long shifts combined with insufficient recovery time can amplify stress and reduce overall well-being among healthcare staff [15,21,22]. The interrelation of occupational type, working hours, and workplace support highlights the multifactorial determinants of QoL. Nurses and employees with extended hours are particularly vulnerable, reinforcing the need for structured policies to reduce occupational stress and promote work-life balance. Prior studies have emphasized that social support, mental health resources, and predictable work schedules are critical for enhancing HCWs’ QoL [22-24].

Our results also show that longer tenure improves QoL, indicating that experience fosters adaptability and professional competence. While it is plausible that increased professional experience enhances adaptability, coping skills, and role competence, thereby improving QoL, it is equally plausible that individuals with better baseline QoL, resilience, and psychological well-being are more likely to remain in employment for longer durations. As healthcare professionals gain experience, they likely develop coping strategies and achieve greater autonomy, which contributes positively to their psychological and physical well-being [17,20]. The negative correlation between working hours and multiple QoL domains emphasizes the importance of regulating shift length. Interventions such as limiting weekly hours, rotating high-intensity duties, and ensuring adequate recovery can enhance social, physical, and psychological well-being among healthcare professionals [15,23]. Mental health support, peer counseling, and mentorship programs are essential to mitigate stress and improve coping strategies in demanding hospital settings [22,25].

Workplace support played a significant role in improving psychological health, consistent with Quinones-Rozo et al. (2024) [23]. Participants who reported supportive work environments exhibited higher psychological health scores, reinforcing the importance of mentorship, leadership recognition, and teamwork in promoting QoL among HCWs. The qualitative evidence from Nalini et al. (2024) and Shivani et al. (2023) corroborates these findings, particularly regarding nurses in demanding specialties [24,25]. Both studies underscore the occupational and psychological strain experienced by nurses, which aligns with our observation of lower QoL scores among nurses with longer working hours. This convergence of quantitative and qualitative data strengthens the validity of our results and highlights the need for targeted occupational interventions.

Limitations

This study was conducted at a single tertiary care hospital, which may limit the generalizability of results to other healthcare settings. The reliance on self-reported measures introduces potential response bias. Additionally, the cross-sectional design precludes establishing causality between occupational factors and QoL. Future longitudinal studies are warranted to evaluate long-term trends, the impact of interventions, and causal relationships between work-related variables and healthcare professionals' well-being.

Implications

Based on these findings, institutions should prioritize policies promoting work-life balance to enhance HCWs’ QoL. Limiting weekly work hours, particularly beyond 48 hours, is essential to safeguard physical and mental health. Additionally, offering mental health support, including counseling services, peer-support programs, and stress management workshops, can help mitigate occupational stress. Workplace interventions should also target staff with lower QoL scores through structured professional development, mentoring, and job management programs. Promoting supportive work environments through cooperative team structures, recognition from supervisors, and leadership engagement is crucial to enhancing mental health, job satisfaction, and overall well-being. Future research should prioritize longitudinal study designs to clarify the temporal and causal relationships between occupational factors, psychosocial stressors, and QoL among HCWs. It should expand beyond single-center settings to include multi-institutional and multi-regional samples, enabling comparison across healthcare systems, staffing models, and resource contexts. This would improve generalizability and allow benchmarking against national or regional norms.

## Conclusions

In conclusion, this study highlights that the QoL among healthcare professionals is significantly influenced by occupation, working hours, length of employment, and workplace support. Nurses and those working more than 48 hours per week reported lower QoL scores across all domains, particularly in physical and social health, underscoring the detrimental impact of long shifts and high occupational demands. Conversely, longer tenure and supportive work environments were associated with better adaptation, coping, and psychological well-being. These findings emphasize the critical need for institutional interventions focused on workload management, work-life balance, mentorship, and mental health support to enhance the overall well-being and job satisfaction of healthcare professionals. The findings of this study underscore the urgent need for systematic workplace planning and structured institutional support to improve the QoL of HCWs. The research underlines the need for structured organizational strategies to improve the physical and mental health of medical workers.

## Appendices

**Table 6:** Full questionnaire used in the study QoL: quality of life; WHOQOL-BREF: World Health Organization Quality of Life - Brief Version; PSS-10: Perceived Stress Scale (10-item version); PSQI: Pittsburgh Sleep Quality Index; AUDIT: Alcohol Use Disorders Identification Test; FTND: Fagerström Test for Nicotine Dependence

Section	Domain	Item No.	Question/Item	Response Options
I	Demographic characteristics	1	Age (in completed years)	Open numeric
		2	Gender	Male/Female/Other
		3	Marital status	Single/Married/Separated/Widowed
		4	Highest educational qualification	Diploma/Graduate/Postgraduate/Doctorate
		5	Years of professional experience	<5/5-10/11-20/>20 years
		6	Designation	Doctor/Nurse
		7	Monthly income (INR)	Categorized ranges
II	Work-related characteristics	8	Department of work	Clinical/Non-clinical (specify)
		9	Current shift pattern	Fixed day/Rotational/Night
		10	Average weekly working hours	<40/40-60/>60
		11	Perceived workload	Low/Moderate/High
III	Perceived Stress (PSS-10)	12	In the last month, how often have you been upset because of something that happened unexpectedly?	0-Never to 4-Very often
		13	Felt unable to control important things in your life?	0-4
		14	Felt nervous and stressed?	0-4
		15	Felt confident about your ability to handle personal problems?*	0-4
		16	Felt that things were going your way?*	0-4
		17	Found you could not cope with all the things you had to do?	0-4
		18	Been able to control irritations in your life?*	0-4
		19	Felt that you were on top of things?*	0-4
		20	Been angered because of things outside your control?	0-4
		21	Felt difficulties were piling up too high to overcome?	0-4
IV	Sleep Quality (PSQI)	22	Subjective sleep quality	Very good → Very bad
		23	Sleep latency (time to fall asleep)	Minutes
		24	Sleep duration	Hours
		25	Sleep efficiency	Derived
		26	Sleep disturbances	PSQI component items
		27	Use of sleep medication	Yes/No
		28	Daytime dysfunction	PSQI component
V	Alcohol Use (AUDIT)	29	How often do you have a drink containing alcohol?	Never → Daily
		30	Typical number of drinks per day	Numeric
		31	Frequency of heavy drinking	Never → Daily
		32-38	Remaining AUDIT items	Standard AUDIT responses
VI	Tobacco Use (FTND)	39	Time to first cigarette after waking	<5 min → >60 min
		40	Difficulty refraining in forbidden places	Yes/No
		41	Cigarette most hated to give up	First in morning/Others
		42	Cigarettes per day	Numeric categories
		43	Smoking frequency morning vs rest of day	Yes/No
		44	Smoking despite illness	Yes/No
VII	QoL - Physical (WHOQOL-BREF)	45-51	Pain, energy, sleep, mobility, activities, work capacity	1-Very poor to 5-Very good
VIII	QoL - Psychological	52-57	Positive feelings, self-esteem, thinking, body image	1-5
IX	QoL - Social relationships	58-60	Personal relationships, support, sexual life	1-5
X	QoL - Environmental	61-68	Safety, finances, health care access, work environment	1-5
XI	Work-life balance & perceptions	69	Overall work-life balance rating	Very poor → Very good
		70	What aspects of your job do you find most rewarding?	Open-ended
		71	What aspects of your work cause the most stress?	Open-ended
